# Editorial: Deep Learning in Brain-Computer Interface

**DOI:** 10.3389/fnhum.2022.927567

**Published:** 2022-05-19

**Authors:** Minkyu Ahn, Sung Chan Jun, Hong Gi Yeom, Hohyun Cho

**Affiliations:** ^1^School of Computer Science and Electrical Engineering, Handong Global University, Pohang-si, South Korea; ^2^School of Electrical Engineering and Computer Science, Gwangju Institute of Science and Technology, Gwangju, South Korea; ^3^Department of Electronics Engineering, Chosun University, Gwangju, South Korea; ^4^Department of Neurosurgery, Washington University School of Medicine, St. Louis, MO, United States

**Keywords:** brain-computer interface, deep learning, machine learning, transfer learning, data augmentation, explainable artificial intelligence

## Introduction

Recent advancements in deep learning with the support of large-scale datasets and computational power have led many studies to adopt deep neural networks (DNNs) to extract features from brain signals and decode brain states, which is an important element in brain-computer interface (BCI). However, several issues remain to be resolved for BCIs to be applicable in the real world. Brain signals are high-dimensional, noisy, and highly nonstationary. In addition, the datasets are limited substantially compared to image data in computer vision fields. Thus, further research that focuses on deep learning (DL) in applications to BCI and a thorough evaluation of the way this application can be used in practice to implement the interface would be beneficial. The primary goal of this Research Topic is to provide an assorted and complementary collection of contributions that show new advancements and review deep learning methods or approaches in BCIs, as well as create a forum for discussion that brings together researchers' contributions to allow progress in deep learning-based BCIs.

## Research Topic Coverage

We collected two reviews and seven research papers on this Research Topic. The authors of the publications accepted presented articles that cover a DL-based BCI for specific applications and a novel model for high BCI performance or transfer learning, data augmentation.

Gutierrez-Martinez et al. conducted a systematic review that covers the current state-of-the-art in visual evoked potential-based BCIs (e.g., P300 or SSVEP-based BCIs) for motor rehabilitation applications and artificial intelligence (AI) algorithms used for detection and classification by analyzing many recent articles. The authors provided an overview of the topic of interest, from traditional machine learning (ML) techniques to cutting-edge DL trends and discussed future challenges in the field.

Ko et al. surveyed the recent advances in short/zero calibration methods in the field of DL-based BCIs. In particular, they provided a good overview of generative model-based and geometric manipulation-based data augmentation methods, and transfer learning techniques that use explicit or implicit methods in DL-based BCIs. The trend in short/zero calibration methods is discussed in detail, and recommendations for the practical use of DL for short or zero calibration BCIs are made for potential users.

Bao et al. proposed a model of a two-level domain adaptation neural network to construct a transfer model for electroencephalography (EEG)-based emotion recognition. The first level uses the maximum mean discrepancy to minimize the distribution discrepancy in deep features from the topological graph of EEG signals, while the second uses the domain adversarial neural network to force the deep features closer to the center of their corresponding class.

Aldayel et al. presented a study on preference detection of a neuromarketing dataset using different combinations of EEG features and different algorithms. The comparison of the algorithms revealed that the deep neural network outperforms k-nearest neighbor (KNN) and support vector machine (SVM) in accuracy, precision, and recall, while Random Forest (RF) achieved similar performance to that of the DNN.

Dolmans et al. presented a novel deep learning model that deals with multimodal data (Galvanic skin response, photoplethysmograms, functional near-infrared spectrograms (fNIRS) and eye movements) to classify perceived mental workload, which is also an interesting and important area in the BCI field.

Borra et al. proposed a novel convolutional neural network (CNN) model, which has a lightweight multi-scale design and guarantees high performance for P300-based BCIs. This model merges the multi-scale temporal learning, which allows a greater decrease in the number of trainable parameters than conventional models and learns multi-scale features as well.

Pei et al. presented a data augmentation method that uses channel-level recombination for motor imagery BCI. To obtain an augmented training set, they divided each sample into two according to the brain region to which the channel belongs and then recombined those samples. Based upon a simulation study, they reported that a CNN model trained with these augmented samples outperforms the typical decoding algorithms.

Kwon and Im proposed a subject-independent CNN-based model for fNIRS-based BCI. This model is designed to be relatively simple, since it uses one-dimensional CNN, but shows reasonable performance in decoding a mental arithmetic state from idle state. The authors tested their model with the typical Linear Discriminant analysis (LDA) and typical CNN model [e.g., EEGNET (Lawhern et al., 2018)] in subject-dependent and independent settings.

Although the typical Transfer Learning approach, which uses known (or labeled) data has shown good performance, the way to build a model that works for unseen data is also of interest. To address this issue, Kostas et al. investigated a self-supervised training approach. Specifically, they adapted techniques and architectures used for language model that show the ability to ingest amounts of data, to EEG analysis. In the study, arbitrary EEG segments were encoded as a sequence of learned vectors, referred to as “BErt-inspired Neural Data Representations”, to determine whether the model is transferable to unseen EEG datasets recorded from unseen subjects, different hardware, and different tasks. The results showed high potential to make an excellent contribution to the BCI field.

All of the articles presented showed important ideas in AI and deep learning approaches in BCIs. The editors are pleased to present this collection of articles to the BCI field and related scientific communities, and hope that it will help researchers advance BCI and its applications.

## Author Contributions

All authors listed have made a substantial, direct, and intellectual contribution to the work and approved it for publication.

## Conflict of Interest

The authors declare that the research was conducted in the absence of any commercial or financial relationships that could be construed as a potential conflict of interest.

## Publisher's Note

All claims expressed in this article are solely those of the authors and do not necessarily represent those of their affiliated organizations, or those of the publisher, the editors and the reviewers. Any product that may be evaluated in this article, or claim that may be made by its manufacturer, is not guaranteed or endorsed by the publisher.
